# The Response of IL-17-Producing B Cells to ArtinM Is Independent of Its Interaction with TLR2 and CD14

**DOI:** 10.3390/molecules23092339

**Published:** 2018-09-13

**Authors:** Patrícia Kellen Martins Oliveira-Brito, Maria Cristina Roque-Barreira, Thiago Aparecido da Silva

**Affiliations:** Departamento de Biologia Celular e Molecular e Bioagentes Patogênicos, Faculdade de Medicina de Ribeirão Preto, Universidade de São Paulo, Ribeirão Preto, SP 14049-900, Brazil; patriciakellen04@hotmail.com (P.K.M.O.-B.); mcrbarre@gmail.com (M.C.R.-B.)

**Keywords:** IL-17, B cells, ArtinM, TLR2, CD14, carbohydrate recognition

## Abstract

ArtinM, a d-mannose-binding lectin from *Artocarpus heterophyllus*, activates antigen-presenting cells by recognizing Toll-like receptor (TLR)2 and cluster of differentiation (CD)14 *N*-glycans, induces cytokine production, and promotes type 1 T helper (Th1) immunity, a process that plays an assisting role in the combat against fungal infections. We recently demonstrated that ArtinM stimulates CD4^+^ T cells to produce interleukin (IL)-17 through direct interaction with CD3. Here, we further investigated the effects of ArtinM on the production of IL-17 by B cell activation. We showed that ArtinM activates murine B cells, increasing IL-17 and IL-12p40 production. The direct effect of ArtinM was sufficient to induce IL-17 production in B cells, and we did not find differences in the levels of IL-17 between the B cells purified from the wild-type (WT) and knockout (KO) mice for TLR2 or CD14 in the presence of ArtinM. Thus, the effects of ArtinM on splenic B cells through carbohydrate recognition may contribute to Th17 immunity; however, the mechanism involved is not associated with the interaction of ArtinM with TLR2 and CD14. The current work represents a pioneering effort in the understanding of the induction of IL-17 by lectins in B cells.

## 1. Introduction

The interleukin (IL)-17 family (IL-17B, IL-17C, IL-17D, IL-17E, and IL-17F) has amino-acid sequence homology and highly conserved cysteine residues; IL-17A was renamed IL-17 [1]. The IL-17 family of cytokines is mainly produced by a distinct lineage of cluster of differentiation (CD)4^+^ T helper (Th) lymphocytes, known as Th17 cells, and the transcription factor RAR-related orphan receptor γT (RORγT) is induced by IL-6 and IL-23 via signal transducer and activator of transcription (STAT)3, which is essential for the Th17 lineage [2]. Th17 polarization can also be induced by low doses of transforming growth factor (TGF)-β together with IL-6. Moreover, the combination of IL-6, IL-1β, and IL-23 is sufficient for inducing the development of Th17 cells in mice [3]. The other cells capable of producing IL-17 are CD8^+^ T cells (also known as Tc17 cells) [4,5], γδ T cells [6,7], invariant natural killer T (iNKT) cells [8,9], natural killer (NK) cells [10,11], and type 3 innate lymphoid cells (ILC3) [12,13]. Recently, Bernejo et al. showed that mature cells of the B lineage act as innate-like producers of IL-17 in response to infection with *Trypanosoma cruzi* [14,15]. This work also demonstrated that splenic B cells do not produce IL-17 upon stimulation with Toll-like receptor (TLR)2, TLR4, or TLR9 ligands; otherwise, the *T. cruzi* trans-sialidase induces IL-17 production by remodeling the glycoproteins on the cell surface of B cells that lack the TLR signal transduction pathway [14,15].

ArtinM, a d-mannose-binding lectin obtained from the seeds of *Artocarpus heterophyllus*, is a homotetramer formed by 16-kDa non-glycosylated subunits [16]. Each polypeptide chain contains a carbohydrate recognition domain (CRD) with affinity for Manα1–3(Manα1–6)Man [17,18]. It is well established that the interaction of ArtinM with TLR2/CD14 *N*-glycans on innate immune cells induces the production of IL-12 and the establishment of Th1 immunity [19,20]. Moreover, our previous study showed that ArtinM promotes the induction of IL-17 release by directly targeting CD4^+^ T cells via an interaction with CD3. Furthermore, we found that ArtinM-induced IL-17 production by spleen cells was inhibited by 30% in the absence of B cells [21]. In addition, we verified that stimulation of murine spleen cells with ArtinM induced IL-17 production at levels significantly higher than those detected in the presence of plant lectins with distinct specificities for sugar recognition [21,22]. These findings reinforce the importance of investigating the capacity of ArtinM to induce IL-17-producing B cells, and the binding of ArtinM to Toll-like receptor (TLR) 2 and CD14 could be relevant, as these receptors are considered crucial for the activation of antigen-presenting cells [19]. In the current work, we found that the direct effect of ArtinM on splenic B cells obtained from C57BL/6 mice is the production of IL-17, and that the presence of TLR2 or CD14 is dispensable for the maintenance of the levels of IL-17 induced by ArtinM.

## 2. Results

### 2.1. Response of IL-17-Producing B Cells in the Presence of ArtinM

**Figure 1:**
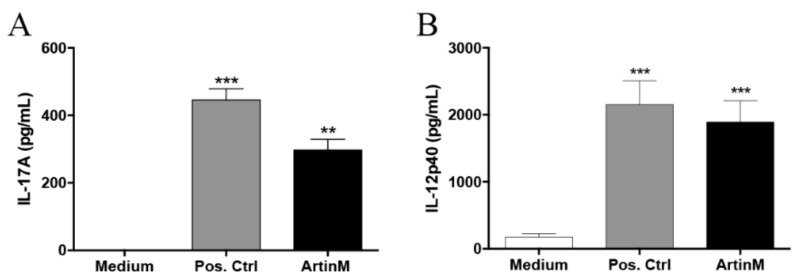
Interleukin (IL)-17- and IL-12-production induced by ArtinM in B cells. Splenic B cells from C57BL/6 mice were purified via magnetic beads, and B cells at a concentration of 2 × 10^6^ cells/mL were distributed in a 48-well plate and incubated for 48 h at 37 °C. The cells were incubated with ArtinM (2.5 µg/mL), a positive control (Pos. Ctrl) of stimulation (IL-6 (10 ng/mL); IL-1β (10 ng/mL); IL-23 (10 ng/mL)), or medium alone (Medium). The levels of IL-17 (**A**) and IL-12p40 (**B**) in the culture supernatants were assessed using ELISA. The values (in pg/mL) obtained for B cells under stimulation were compared with those of the cells in the medium. Data are shown as the mean ± SD; ** *p* < 0.01 and *** *p* < 0.001 were determined using the Kruskal–Wallis test followed by the Dunn’s multiple comparison test.

Previously, we identified that ArtinM acts on CD4^+^ T cells to induce IL-17 production via CD3 recognition [21]. Moreover, the IL-17 production induced by ArtinM in spleen cells was impaired after B-cell depletion [21]. Thus, we assayed the ArtinM activity in splenic B cells, selected with magnetic beads, and we evaluated the IL-17 production in the culture supernatant using ELISA after 48 h of incubation at 37 °C. High levels of IL-17 were detected after ArtinM stimulation, compared to the negative control (medium), and stimulation with a cytokine cocktail of IL-6 (10 ng/mL)/IL-1β (10 ng/mL)/IL-23 (10 ng/mL) was considered as the positive control (Pos. Ctrl) (Figure 1A). Considering that ArtinM induces high levels of IL-12p40 by antigen-presenting cells via TLR2/CD14 interaction, and that this cytokine can be produced by B cells in response to TLR-agonist stimulation, we also measured the levels of IL-12 released by B cells after 48 h of stimulation with ArtinM. We found that the production of IL-12p40 increased significantly upon ArtinM stimulation, compared to the cells in the medium (Figure 1B). Therefore, the direct effect of ArtinM was sufficient to induce IL-17- and IL-12p40-production in B cells, thus contributing to the development of Th17 and Th1 cells, respectively.

### 2.2. IL-17 Production Induced by ArtinM in B Cells Is Not Dependent on TLR2 and CD14 Recognition

**Figure 2:**
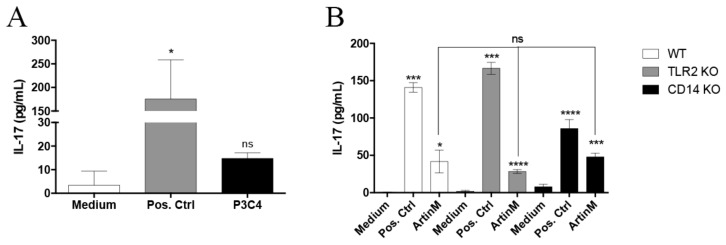
Effect of the absence of TLR2 or CD14 on IL-17 production induced by ArtinM in B cells. B cells (1 × 10^6^/mL) from wild-type (WT), TLR2 knockout (KO), and CD14 KO mice were stimulated with ArtinM (2.5 µg/mL), P3C4 (1 µg/mL), a positive control of stimulation (IL-6 (10 ng/mL); IL-1β (10 ng/mL); IL-23 (10 ng/mL)), or medium alone (Medium) for 48 h at 37 °C. (**A**) IL-17 production was measured using ELISA of the culture supernatant of B cells incubated with P3C4, the positive control of stimulation, or medium. (**B**) WT, TLR2 KO, and CD14 KO mice were used to obtain splenic B cells that were stimulated with ArtinM, the positive control, or medium. The culture supernatants were used for the measurement of IL-17 using ELISA, and the cells under stimuli were compared with the cells in the medium; the values were also compared between the WT and TLR2 KO or CD14 KO B cells stimulated with ArtinM. Data are shown as the mean ± SD; * *p* < 0.05, *** *p* < 0.001, **** *p* < 0.0001 and not significant (ns) were determined using the Kruskal–Wallis test, followed by the Dunn’s multiple comparison test.

The immunomodulatory activity of ArtinM on macrophages involves a mechanism that is crucially dependent on TLR2 and the co-receptor CD14 [19,20]. As the TLR pathway can be responsible for the production of pro-inflammatory cytokines in B cells, the interaction of ArtinM with TLR2/CD14 was proposed as the major mechanism related to IL-17 production by B cells, stimulated with ArtinM. Initially, we investigated the capacity of a TLR2 agonist (P3C4) to promote IL-17 production by B cells from wild-type (WT) mice, and the P3C4 stimulation was not sufficient to induce high levels of IL-17 in B cells compared to the cells in the medium (Figure 2A). The cytokine cocktail of IL-6 (10 ng/mL)/IL-1β (10 ng/mL)/IL-23 (10 ng/mL) was considered as the positive control (Pos. Ctrl) (Figure 2A). In Figure 2B, we obtained splenic B cells from wild-type (WT) mice and knockout (KO) mice for TLR2 or CD14, and we compared the IL-17 levels produced by these B cells in the presence of ArtinM. Interestingly, IL-17 production by B cells of WT mice in response to ArtinM was not significantly different to that found in TLR2 KO or CD14 KO mice under the same conditions (Figure 2B). These findings demonstrate that the TLR2/CD14 recognition by ArtinM is not critical for the induction of IL-17 production in B cells.

## 3. Discussion

The known recognition of TLR2/CD14 *N*-glycans on innate immune cells by ArtinM is essential for inducing Th1 cytokine production, which favors the host immune response against infection by intracellular pathogens [19,23,24,25,26,27,28]. Otherwise, the ArtinM interaction with the CD3 receptor is associated with the induction of IL-17 in CD4^+^ T cells, and we also observed that ArtinM induces antigen-presenting cells (APCs) to express IL-23 and IL-1, which are known to positively influence IL-17 production by CD4^+^ T cells [21]. In this way, we verified that the spleen cell suspensions collected after the depletion of B cells showed a 30% inhibition of IL-17 production after ArtinM stimulation [21]. Then, in the current work, we evaluated the effect of ArtinM on splenic B cells associated with the induction of IL-17, and the activity of lectin was able to promote the release of IL-17 from B cells. Interestingly, the recognition of TLR2 or CD14 by ArtinM was not essential for the maintenance of the level of IL-17, induced by the lectins in B cells.

Previous studies in mice clearly showed that IL-17 has a major role in the host defense against experimental infections of the oral cavity, skin, intestine, lungs, and vagina, of which bacterial and fungal infectious diseases are the main sources [29]. In contrast, the IL-17 family and its receptors are well understood in the context of autoimmunity, autoinflammation, and allergies, which suggests that the balance between pathogenic and protective IL-17 immunity is essential in the application of therapeutic strategies for inflammatory and infectious diseases [29]. Hence, IL-17-producing B cells became an important field of research, based on the pioneering study published by Bermejo et al. (2013) [14,15]. These authors described that B cells are the principal source of IL-17 after infection with *T. cruzi*, and that direct exposure to *T. cruzi*, or to the trans-sialidase enzyme from this parasite, is responsible for the induction of IL-17 production in B cells [14,15]. Our group has been investigating the direct effect of ArtinM on murine T cells in order to understand the mechanisms involved in the immunomodulatory activity triggered by ArtinM [21,22,30,31], and the capacity of ArtinM to induce IL-17 production in spleen cells and CD4^+^ T cells was recently reported [21]. Interestingly, we found reduced levels of IL-17 induced by ArtinM in spleen cells after B-cell depletion [21], and the polyclonal activation of B cells induced by ArtinM was verified in naïve mice that received ArtinM at various doses [32]. Thus, these findings suggest the involvement of B cells in the immunomodulatory activity induced by ArtinM. The current work on IL-17 production induced by ArtinM in B cells reinforced our hypotheses.

Bermejo et al. described that B cells use alternative IL-17-triggering signals, as demonstrated in infected mice deficient in the receptor for IL-23 (IL-23R) or deficient in IL-6. Moreover, these authors observed that B-cell-T-cell cooperation through the co-stimulatory receptor CD40 pathway was also not required [14,15]. Additionally, the direct exposure of B cells to *T. cruzi* trypomastigotes or *T. cruzi* trans-sialidase drives the formation of IL-17^+^ B cells via CD45- and Btk-dependent signaling [14,15]. We found that the interaction of ArtinM with B cells was also sufficient to induce high levels of IL-12p40 as observed in innate immune cells, while IL-6 production by B cells was not detected upon ArtinM stimulation (data not shown). These results show that ArtinM induces IL-17 production in B cells in an IL-6-independent manner, and that IL-6 is essential for the development of Th17 cells in mice. Our results, therefore, suggest that IL-17 production in B cells is not linked to high levels of IL-6. Further studies should address the participation of the CD45 receptor in the effect of ArtinM on B cells because of the high content of *N*-glycans in this receptor [33], which can be recognized by lectin in order to mediate B-cell activation.

Although TLRs can promote IL-17 production by innate cells, the induction of the TLR signaling pathway in B cells by TLR2, TLR4, and TLR9 ligands did not promote a significant increase in IL-17 production [14,15]. We also observed that the TLR2 agonist P3C4 was insufficient to induce IL-17 production in splenic B cells, as reported by Bermejo et al. Considering that the interaction of TLR2 and CD14 with ArtinM on the surface of antigen-presenting cells is required to induce immunomodulatory activity [19,20], we assayed IL-17 production in B cells obtained from knockout mice for TLR2 or CD14 in response to ArtinM, and found that the levels of IL-17 were not different between the B cells from the WT and KO mice. Surprisingly, purified B cells in vitro under direct exposure to ArtinM released high levels of IL-17 in a TLR2/CD14-independent manner. Thus, our hypothesis that ArtinM targets CD45 *N*-glycans on B cells, triggering Src and Btk kinase intracellular signaling, is substantially relevant, and further studies are required to demonstrate this mechanism.

In conclusion, ArtinM stimulates the production of IL-17 by B cells, and the mechanism involved is not associated with the interaction of ArtinM with TLR2 and CD14. The present study demonstrates that ArtinM, through carbohydrate recognition on splenic B cells, can contribute to Th17 immunity. Our work is pioneering in the induction of IL-17 by lectins in B cells, and illustrates new strategies to modulate immunity.

## 4. Materials and Methods

### 4.1. Ethics Statement

The Committee of Ethics in Animal Research of the College of Medicine of Ribeirão Preto at the University of São Paulo approved the animal experiments. The protocol no. 54/2016 was conducted in accordance with the Ethical Principles in Animal Research, adopted by the Brazilian College of Animal Experimentation.

### 4.2. Animals

C57BL/6 (WT), TLR2 (C57BL/6 genetic background), and CD14 KO (C57BL/6 genetic background) mice (6–8 weeks old) were used in this study. The mice were acquired from the animal facility of the Ribeirão Preto Medical School at the University of São Paulo, and were bred and housed under optimized hygienic conditions at the animal facility of the Molecular and Cellular Biology Department of the Ribeirão Preto Medical School. The mice were anesthetized with xylazine (125 mg/kg) and ketamine (10 mg/kg) in phosphate buffer solution (PBS), with their neck hyperextended and their trachea exposed after incision.

### 4.3. ArtinM Lectin and B-Cell Isolation

ArtinM was purified as described previously [16] from the saline extract of *Artocarpus heterophyllus* (jackfruit) seeds through affinity chromatography with immobilized carbohydrate columns. Before use, the ArtinM aliquots were incubated for 1 h with a polymyxin solution (50 µg/mL; Sigma-Aldrich, St. Louis, MO, USA).

Suspensions of the spleen cells, obtained from two mice, were prepared as reported by da Silva et al. [30]. The obtained cell suspensions were used to isolate B cells using the Pan B Cell Isolation Kit for mice from Miltenyi Biotec (Auburn, CA, USA), according to the manufacturer’s instructions. The purification of B cells was performed for determination of IL-17 and IL-12p40 production in response to ArtinM. Afterward, B cells were isolated from WT, TLR2 KO, and CD14 KO mice to measure the production of IL-17 induced by ArtinM.

### 4.4. Measurement of the Cytokines

B cells (1 × 106/mL or 2 × 106/mL) were cultured in Roswell Park Memorial Institute (RPMI) 1640 (containing 10% fetal bovine serum) for 48 h under stimulation with ArtinM (2.5 µg/mL), palmitoyl-3-cysteine-serine-lysine-4 Pam3CSK4 (P3C4, 1 µg/mL; Sigma-Aldrich), a mixture (positive control—Pos. Ctrl; PeproTech, Rock Hill, NJ, USA) of IL-6 (10 ng/mL) plus IL-1β (10 ng/mL) and IL-23 (10 ng/mL), or medium alone (Medium). After 48 h of incubation, the B cells were centrifuged (300× g, 10 min at 24 °C), and the supernatants were collected for measurement of IL-17 (Ready-SET-Go!^®^ Kit; Cat. No. 88-7371, Fisher Scientific, Hampton, NH, USA) and IL-12p40 levels by means of an enzyme-linked immunosorbent assay (ELISA) using the OptEIA kit (BD Biosciences, Franklin Lakes, NJ, USA), according to the manufacturer’s instructions.

### 4.5. Statistical Analysis

Results are presented as the mean ± standard deviation (SD), and all data were analyzed using Prism 6.0 (GraphPad Software Inc., La Jolla, CA, USA). All statistical determinations for normality were analyzed by the Kolmogorov–Smirnov test with the Dallal–Wilkinson–Lilliefor *p*-value, and when the distribution could not be assumed to be normal, the Kruskal–Wallis test was used. Statistical determinations of the differences in means between groups were performed by the Kruskal–Wallis test, followed by the Dunn’s multiple comparison test. Differences that provided *p* < 0.05 were considered statistically significant. All experiments were performed in triplicate and were repeated three times.
